# The Evaluation of Change in Choroidal Circulation Time before and after Half-Dose Photodynamic Therapy in Central Serous Chorioretinopathy Using Wide-Field Indocyanine Green Angiography

**DOI:** 10.3390/jcm13144257

**Published:** 2024-07-21

**Authors:** Ruri Sugiyama, Ryusaburo Mori, Akiyuki Kawamura, Koji Tanaka, Hajime Onoe, Yu Wakatsuki, Hiroyuki Nakashizuka

**Affiliations:** Nihon University School of Medicine, Nihon University Hospital, 1-6 Kanda-Surugadai, Chiyoda-ku, Tokyo 101-8309, Japan; yamamoto.ruri@nihon-u.ac.jp (R.S.); mori.ryusaburo@nihon-u.ac.jp (R.M.); kw-eye-c@xb3.so-net.ne.jp (A.K.); tanaka.koji@nihon-u.ac.jp (K.T.); onoe.hajime@nihon-u.ac.jp (H.O.); wakatsuki.yu@nihon-u.ac.jp (Y.W.)

**Keywords:** central serous chorioretinopathy, photodynamic therapy, indocyanine green angiography

## Abstract

**Background**: Indocyanine green angiography (ICGA) is often used for diagnosis of, and as an indication to apply laser treatment for, central serous chorioretinopathy (CSC). Although photodynamic therapy (PDT) is effective against CSC, the details of the mechanism are unknown. To verify the effect of PDT, we compared the time of choroidal circulation before and after PDT in CSC patients, using ICGA. **Methods**: Seven eyes of seven patients (six male, one female) who were diagnosed with chronic CSC associated with serous retinal detachment (SRD) in the macular area and who underwent half-dose PDT were included. Wide-field ICGA images with an angle of 102° were taken and evaluated at the superior and inferior temporal quadrants. Choroidal circulation time (CCT) was defined as the time from the start of contrast filling in the choroidal artery to the first appearance of contrast filling in the temporal vortex vein ampulla. **Results**: The average CCT before and after PDT in the superior temporal vortex vein was 3.96 s and 5.41 s (*p* = 0.018), and 4.12 s and 5.02 s (*p* = 0.046) in the inferior temporal vortex vein, respectively. All SRD and choroidal vascular hyperpermeability areas dissolved after PDT. **Conclusions**: In this pilot study, half-dose PDT prolonged CCT in CSC patients, indicating the effect of selective vascular obstruction in the choriocapillaris.

## 1. Introduction

Central Serous Chorioretinopathy (CSC) is a common retinal disease with a mean incidince rate of 54.2 per 100,000 person-years in men and 15.7 per 100,000 person-years in woman in Japan, showing higher prevalence in men [1]. CSC is characterized by leakage from the retinal pigment epithelium (RPE) leading to serous retinal detachment (SRD) [2]. Presence of SRD is known to be related to decreased visual acuity, and longer term of SRD can cause worse visual acuity associated with retinal atropthy [3]. Angiography not only plays an important role in diagnosis, but is also used as an indicator for laser treatment against CSC. Indocyanine green angiography (ICGA) shows choroidal vascular hyperpermeability (CVH) [4], indocyanine green dye leakage [5] and dilated choroidal vessels which are known as pachyvessels, which highly contribute to the diagnosis of CSC. ICGA is also necessary for managing CSC since photodynamic therapy (PDT) is generally applied to areas showing CVH [6]. 

There are many reports showing positive results of PDT for CSC cases which had no indication of laser treatment. Laser treatment is associated with the risk of scotoma, so there are certain cases where PDT is preferable to laser treatment depending on the location of the leakage. However, the standard PDT protocol had risks of side-effects, such as pigmentary changes and atrophy of the RPE [7]. To reduce complications, PDT has been conducted by reducing the dosage, fluence, and time of laser radiation. Recently, thinning of the subfoveal choroid of the posterior pole, including the areas outside the arcade vessels after reduced fluence-PDT were reported [8]. Funatsu et al. also reported that choroidal thickness of all areas within an angle view of 100°, measured by ultra-widefield optical coherence tomography (OCT), decreased after PDT [9]. 

After half-dose PDT, the choroidal blood flow of the irradiated area was reduced, as examined by laser speckle flowgraphy [10,11]. Spaide et al. suggested that intervortex vein anastomosis may play a role in the pathogenesis of CSC, and imaging at a wider angle has enabled the evaluation of the hemodynamics of the peripheral retina [12]. Bacci et al. also performed ultra-widefield ICGA on pachychoroid patients and reported that CVH are likely to be seen in temporal quadrants, and were prominent in larger vortex vessel drainage areas [13].

Since the mechanism of PDT against CSC is still unclear, we decided to evaluate the effect of PDT by using wide-field ICGA. To the best of our knowledge, this is the first report to compare the time of circulation in the choroid using wide-field ICGA before and after treatment of half-dose PDT. In the present study, we evaluated the change in time of choroidal circulation before and after PDT treatment.

## 2. Materials and Methods

Seven eyes of 7 patients (6 male, 1 female) who were diagnosed with CSC associated with SRD in the macular area and who underwent half-dose PDT during the period of November 2021 to January 2022 in Nihon University Hospital were reviewed as a pilot study. Average age of the participants was 48.7 ± 5.68 years. These patients had leakage spots near the macular area, so PDT was chosen since laser treatment has the possibility of inducing scotoma in the central visual field. The average spherical equivalent was −2.43 ± 2.62 diopter.

All 7 patients were diagnosed with chronic CSC, which had a clinical course of more than 6 months. All participants underwent tests of best correct visual acuity (BCVA), intraocular pressure, color fundoscopy, autofluorescence, and fluorescence angiography. Wide-field ICGA and OCT were taken before and 1 month after treatment. The exclusion criteria were patients with a history of PDT, focal photocoagulation or subthreshold micro-pulse laser photocoagulation, and patients with a history of uveitis or other ocular diseases causing macular neovascularization and secondary SRD.

PDT was performed using half-dose verteporfin (Visudyne; Clinigen K.K, Staffordshire, UK). A total of 3 mg/m^2^ of verteporfin was infused over 10 min. Laser treatment began 15 min after the beginning of infusion. The total light energy delivered to the hyperpermeability area was 50 J/cm^2^. The area of irradiation was set to cover the hyperfluorescent area associated with CVH, measured in images of the late phase of ICGA.

All 7 patients included in this study were chronic CSC patients. The primary endpoint was the difference in choroidal circulation time (CCT) before and after PDT. ICGA imaging was performed using Spectralis HRA + OCT (HEYEX Ver.1.10.2.0 AQM Ver.6.9.4.0 Viewer Ver. 6.9.5.0) (Heidelberg Engineering, Heidelberg, Germany). The images were taken from the early phase to the late phase with the wide-field lens attached, which expands the filming angle to 102°.

### Evaluation of Choroidal Circulation Time (CCT)

The dynamic angiographic filling sequence was recorded at 8.8 frames per second. CCT was defined as the time from the start of contrast filling in the choroidal artery to the first appearance of contrast filling in the vortex vein ampulla. ICGA video was separated into still images, and the time was measured by 2 authors (R.S. and R.M.). If there was a discrepancy in the reading for even one image, a third specialist (A.K) evaluated the CCT. The early choroidal arterial phase had to be filmed at a single angle, and due to the angle of the camera, it was difficult to include both superior and inferior vortex veins of the nasal and temporal side. Therefore, all images included both superior and inferior temporal vortex veins. The circulation time post-treatment was subtracted from pre-treatment time. The circulation time in the superior temporal vortex vein and the inferior temporal vortex vein was measured separately. 

As a secondary endpoint, the relationship between the irradiation size and the circulation time was evaluated. Additionally, the change in choroidal thickness, SRD, CVH, the size of the hypofluorescence area in the early phase and 5 min after the beginning of contrast enhancement, between pre-treatment and post-treatment, was also evaluated.

Statistical analysis was performed using SPSS version 23.0 (IBM Japan, Ltd., Tokyo, Japan). Wilcoxon signed-rank test was used to compare the choroidal circulation time before and after PDT. Spearman’s rank correlation test was used to evaluate the correlation between the size of the radiation area and the degree of extension of the circulation time due to treatment. A *p*-value less than 0.05 was considered statistically significant.

## 3. Results

### 3.1. Change in CCT before and after PDT

The circulation time, difference between before- and after-PDT values, and the characteristics of each case are listed on Table 1.

**Figure 1:**
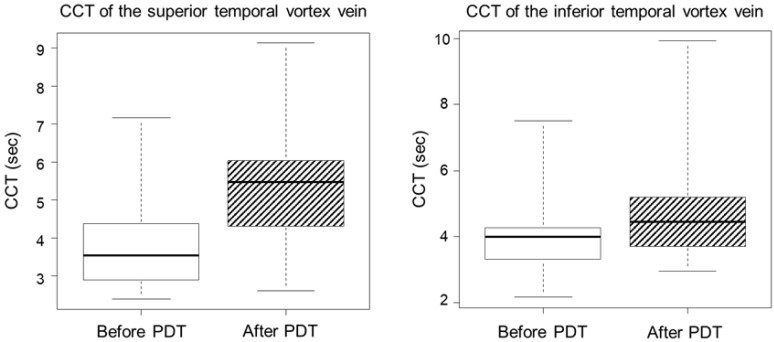
CCT was significantly prolonged after PDT in both superior (**left box plot**) and inferior (**right box plot**) temporal vortex veins (superior temporal vortex vein: *p* = 0.018, inferior temporal vortex vein: *p* = 0.046). CCT, choroidal circulation time; PDT, photodynamic therapy.

In superior temporal vortex veins, the average CCT was 3.96 ± 1.65 s at pre-treatment, and 5.41 ± 2.04 s at post-treatment (*p* = 0.018). In inferior temporal vortex veins, the average CCT at pre- and post-treatment was 4.12 ± 1.68 s and 5.02 ± 2.32 s, respectively (*p* = 0.046). The average of the differences in CCTs between before and after PDT of the superior and inferior temporal vortex veins were 1.46 ± 0.82 s and 0.90 ± 0.84 s, respectively. CCT was significantly extended after PDT in both superior and inferior temporal vortex veins (Figure 1).

### 3.2. Secondary Endpoint

The relationship between the size of the irradiation area and CCT was also evaluated. The average size of radiation was 4928 ± 1644 µm. Spearman’s rank correlation was calculated to assess the relationship between the radiation size and CCT. There was a possibility of positive correlation between radiation size and CCT in inferior vortex veins (ρ = 0.714); however, correlation was not significant in superior temporal vortex veins (ρ = 0.429). SRD in all seven cases dissolved after PDT. The average subfoveal choroidal thickness was 376 ± 82.6 µm before PDT, and 301 ± 77.0 µm after PDT. The size of the hypofluorescent area in the early stage and 5 min after contrast enhancement in ICGA between pre-treatment and post-treatment were different in all cases. The hypofluorescent areas seen 5 min after injection at post-treatment corresponded to the area of radiation size in PDT in all cases. CVH was seen in six cases before PDT and two cases after PDT. All CVH areas that were included in the radiation area dissolved.

### 3.3. Representative Case

**Figure 2:**
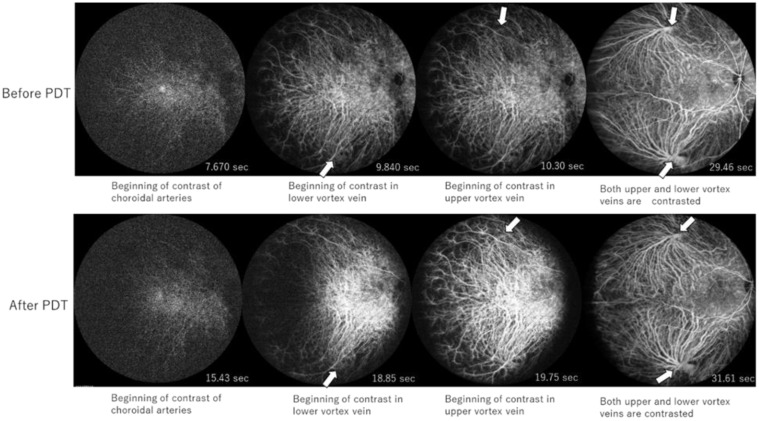
Images of wide-field indocyanine green angiography before and after photodynamic therapy in the representative case. The arrows indicate the dye appearance of the vortex veins. CCT before and after PDT of the superior temporal vortex vein was 2.63 (10.3 − 7.67 = 2.63) and 4.32 (19.75 − 15.43 = 4.32) seconds, respectively. In inferior temporal vortex veins, CCT before and after PDT was 2.17 (9.84 − 7.67 = 2.1) and 3.42 (18.85 − 15.43 = 3.42) seconds, respectively. PDT, photodynamic therapy.

**Figure 3:**
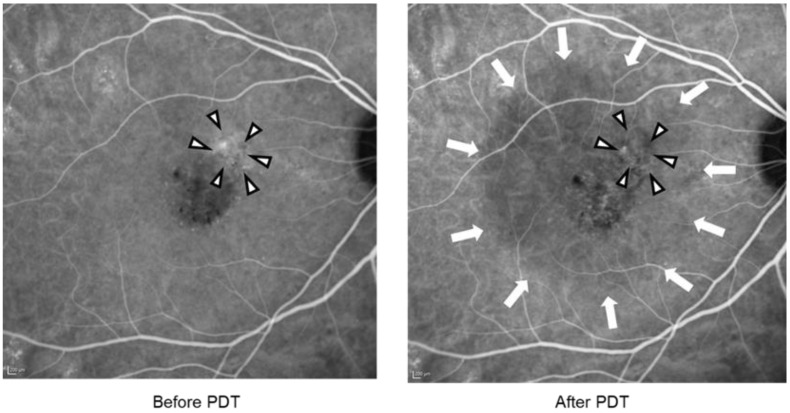
Indocyanine green angiography images before (**left**) and after (**right**) photodynamic therapy. CVH within the irradiation area regressed after treatment (arrowheads). The size of the hypofluorescent area expanded after PDT and matched the size of the irradiation area (arrows). PDT, photodynamic therapy.

**Figure 4:**
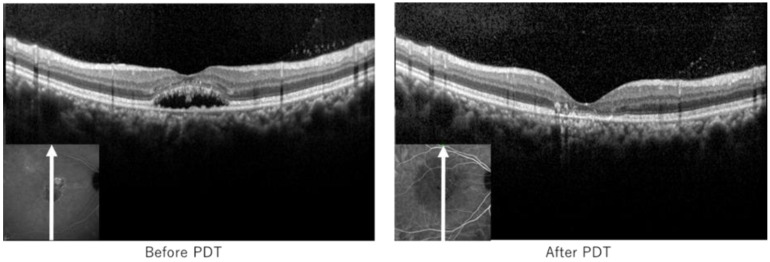
Optical coherence tomography before and after photodynamic therapy of the representative case. SRD regressed completely 1 month after PDT. PDT, photodynamic therapy.

A patient in her forties was diagnosed with chronic CSC (Case 5). CCT before and after PDT of the superior temporal vortex vein was 2.63 and 4.32 s, respectively. In inferior temporal vortex veins, CCT before and after PDT was 2.17 and 3.42 s, respectively (Figure 2). Thus, the changes in CCT were +1.69 s and +1.25 s in the superior and inferior temporal vortex veins, respectively. CVH in the upper nasal area of the macula disappeared, and the hypofluorescent area in ICGA was seen after PDT. The size of the hypofluorescent area was different from the area before PDT (Figure 3). Furthermore, SRD regressed after PDT as well (Figure 4).

## 4. Discussion

In the present study, we compared the CCT between pre-treatment and post-treatment using wide-field ICGA to consider the mechanism of PDT against CSC. The fact that the circulation time was prolonged after treatment in both superior and inferior temporal vortex veins showed that blood flow obstruction caused by PDT extended to the peripheral area of the retina. Thinning of the choroidal thickness in and around the irradiated area was also reported [8], and considering the theorem of fluid dynamics Q = A · V (Q: blood flow volume, A: sectional area of vessel, V: blood flow velocity) [14] and the decrease in luminal areas in the choroid after PDT [15], the prolonged circulation time in this study suggests that perfusion volume was reduced after PDT treatment.

Reducing the dose of verteporfin by half can decrease the complications associated with standard-dose PDT. Therefore, the efficacy of half-dose PDT has been widely reported. Even if PDT is performed at half-dose, a decrease in choroidal blood flow after PDT has also been reported [10]. In this report, the choroidal blood flow was significantly reduced depending on the dosage of verteporfin. In our study, PDT reduced the increased choroidal circulation volume leading to extension of CCT, and also revealed the possibility of a correlation between the size of the irradiation area and CCT. In other words, CCT may be extended depending on the size of the radiation of PDT. It is presumed that wider irradiation areas provoke wider areas of circulatory disturbance, which delays blood inflow to the vortex veins.

All hypofluorescent areas seen 5 min after an ICG injection at post-treatment matched the size of the radiation area of PDT. According to the report by Schlotzer-Schrehardt et al., in donor eyes with melanoma, selective vascular occlusion in the choriocapillaris was seen after PDT and there was no occlusion in the vessels of the outer layer of the choroid [16]. Thus, the hypofluorescent areas seen in all seven cases after PDT in this study were thought to be blood flow obstructions of the choriocapillaris. Extension of CCT was observed presumably because of selective occlusion of the vessels in the choriocapillaris, causing a decrease in blood flow of the peripheral vessels, such as choroidal and vortex veins.

CVH was first reported by Guyer et al. in 1994 as the cause of CSC [4], and since then, there have been reports indicating that CVH can also be seen in the fellow eyes of unilateral CSC patients, and even in healthy eyes [17]. The detailed pathophysiology of CVH is not fully understood; however, it is known that PDT reduces the diameter of choroidal vessels and vascular hyperpermeability in CSC [18]. This is consistent with the fact that all CVH areas regressed after PDT in this study. CSC is thought to be caused by barrier function disorder associated with the elevation of choroidal hydrostatic pressure [10]. The obstruction in choriocapillaris seen after PDT may cause the filling delay in ICGA in the peripheral vessels, showing the possibility that volume overload against the choroid was removed by PDT, leading to a decrease in CVH, and resulting in SRD regression.

There are several limitations to this study. First, although this study showed a delay in CCT after PDT, we have not assessed outcomes more than 1 month after treatment. Therefore, it is not clear if this change is only temporary, or if it is persistent. We have previously reported that IR images showed improvement over time after PDT [19], and it will be necessary to follow up for a longer period of time and assess whether the findings change as time proceeds in the current patient cohort as well. Second, the control group was not established since patients with normal eyes could not undergo ICGA for ethical reasons. Third, the gender ratio of the studied patients was unbalanced. Fourth, during PDT, laser radiation was applied to the retina in a circular pattern including hyperfluorescent areas suggesting CVH; therefore, the radiation areas deviated from the center of the macula in some cases. In CSC patients, cases with asymmetric watersheds are reported to be frequent [20], and these facts may affect the circulation time of the vortex veins. Fifth, in this study, the average spherical equivalence was myopic, but the choroidal thickness of all cases was not thinned despite the myopic refraction error. Even so, assessing the circulation time of emmetropic and hypermetropic eyes is necessary. However, this is the first report comparing the CCT before and after PDT, and is valuable in understanding the mechanism of PDT treatment for CSC. Moreover, the number of cases where we were able to measure the circulation time was small. Finally, since the angle of view was limited, the CCT of vortex veins was only measured on the temporal side, and not on the nasal side. Due to these limitations, further evaluation is required in the future.

## 5. Conclusions

In conclusion, this pilot study demonstrated that selective vascular obstruction in the choriocapillaris of the radiation area by half-dose PDT prolonged CCT.

## Figures and Tables

**Table 1:** The characteristics of the irradiation area, subfoveal choroidal thickness, and choroidal circulation time of the superior and inferior temporal vortex veins in each case. The size of the irradiation area is considered by the greatest linear dimension. CCT, choroidal circulation time; PDT, photodynamic therapy.

		Subfoveal Choroidal Thickness (µm)		CCT of the Superior Vortex Vein			CCT of the Inferior Vortex Vein		
Case	Irradiation Area (µm)	Before PDT	After PDT	Before PDT	After PDT	Difference between before and after PDT	Before PDT	After PDT	Difference between before and after PDT
1	5000	498	341	2.40	2.62	0.22	2.97	2.96	−0.01
2	3000	374	350	3.77	4.33	0.56	3.99	3.99	0
3	4000	293	197	3.19	5.46	2.27	4.32	4.9	0.58
4	3000	392	347	5.01	6.27	1.26	3.65	4.45	0.8
5	6000	321	304	2.63	4.32	1.69	2.17	3.42	1.25
6	6500	467	377	3.54	5.81	2.27	4.22	5.47	1.25
7	7000	289	188	7.17	9.12	1.95	7.51	9.92	2.41
Average	4928	376	301	3.96	5.42	1.46	4.12	5.02	0.90

## Data Availability

Data is contained within the article. Further data that support the findings of this study are available from the corresponding author upon reasonable request.
